# A conceptual framework for the phylogenetically constrained assembly of microbial communities

**DOI:** 10.1186/s40168-019-0754-y

**Published:** 2019-10-30

**Authors:** Daniel Aguirre de Cárcer

**Affiliations:** Departamento de Biología, Universidad Autónoma de Madrid, Madrid, Spain

**Keywords:** Community assembly, Microbiome, 16S, Community ecology

## Abstract

Microbial communities play essential and preponderant roles in all ecosystems. Understanding the rules that govern microbial community assembly will have a major impact on our ability to manage microbial ecosystems, positively impacting, for instance, human health and agriculture. Here, I present a phylogenetically constrained community assembly principle grounded on the well-supported facts that deterministic processes have a significant impact on microbial community assembly, that microbial communities show significant phylogenetic signal, and that microbial traits and ecological coherence are, to some extent, phylogenetically conserved. From these facts, I derive a few predictions which form the basis of the framework. Chief among them is the existence, within most microbial ecosystems, of phylogenetic core groups (PCGs), defined as discrete portions of the phylogeny of varying depth present in all instances of the given ecosystem, and related to specific niches whose occupancy requires a specific phylogenetically conserved set of traits. The predictions are supported by the recent literature, as well as by dedicated analyses. Integrating the effect of ecosystem patchiness, microbial social interactions, and scale sampling pitfalls takes us to a comprehensive community assembly model that recapitulates the characteristics most commonly observed in microbial communities. PCGs’ identification is relatively straightforward using high-throughput 16S amplicon sequencing, and subsequent bioinformatic analysis of their phylogeny, estimated core pan-genome, and intra-group co-occurrence should provide valuable information on their ecophysiology and niche characteristics. Such a priori information for a significant portion of the community could be used to prime complementing analyses, boosting their usefulness. Thus, the use of the proposed framework could represent a leap forward in our understanding of microbial community assembly and function.

## Introduction

Microbes represent most of the Earth’s biodiversity and a large fraction of its biomass and play essential and preponderant roles in ecosystem processes [1]. While in nature microorganisms normally appear as communities of genetically distinct populations (i.e., microbial communities. Table 1, the rules that govern their assembly are still poorly understood [10], despite the actual consensus that such knowledge would greatly improve our ability to understand and manage microbial communities [11, 12], positively impacting, for instance, agriculture [13] and healthcare [14].

**Table 1:** Important terms and definitions employed

Term	Definition	Reference/adapted from
Community assembly	The sum of all processes that shape the composition of a microbial community	[2]
Dispersal	Movement of organisms across space	[2]
Diversification	Increase in diversity of populations in a community caused by the emergence of new genetic variants	[2]
Drift	Stochastic changes in the relative abundance of populations in a community over time	[2]
Ecological coherence	Shared life strategy or traits among a group of populations that distinguish them from members of other groups	[3]
Ecological function	A population’s interaction or ecological role that prevents secondary extinctions, maintains a biogeochemical flux or pool, or supports ecosystem productivity.	[4]
Higher-scale sampling	Refers to the common inability to sample individual patches from a microbial environment	This study
Metacommunity	A set of local communities linked by dispersal of multiple interacting species	[5]
Metacommunity theory	The study of spatially distinct communities linked through dispersal	[6]
Microbial community	Group of potentially interacting microbial populations that co-exist in space and time	[7]
Microbial regimes	Alternative functional states operating in apparently equal environments	This study
Non-phylo-niches	Within a patch, niches whose occupancy requires a specific set of traits not showing strong phylogenetic conservation	This study
Patch	A locality capable of holding a local community	[6]
Phylo-niches	Within a patch, niches whose occupancy require a specific phylogenetically conserved set of traits	This study
Phylogenetic core groups (PCGs)	Discrete portions of the phylogeny present in all instances of a given microbial regime	This study
Population	All genetically and functionally homogeneous individuals within a patch	This study
Priority effect	Ability of early arriving species to competitively suppress late-arriving ones	[8]
Selection	Changes in community composition caused by deterministic fitness differences between populations	[2]
Trait	Any heritable characteristic that affects the fitness or function of an individual	[9]

Our knowledge of microbial communities’ composition, distribution, and to a certain extent function has greatly increased over the last decade, chiefly thanks to the advent of high-throughput sequencing technologies. Significantly, large microbial community composition inventories have been generated from a myriad of microbial ecosystems [12]. However, its study has been often descriptive in nature, technology-centered and, somewhat, wanting in the formal use of hypotheses. In this sense, we lag behind on the identification of broadly applicable principles that can help us understand microbial community assembly [15, 16], and the development of conceptual frameworks with which to study such complex systems.

In this regard, the past few years have seen the progressive recognition of the role that Vellend’s synthesis of community ecology [2] can play in our understanding of microbial community assembly [7, 17, 18], with an ever-increasing number of studies employing its four basic ecological processes (drift, dispersal, selection, and diversification; Table 1) to examine community assembly and dynamics (e.g., [19, 20]). Stegen et al. have delineated the theoretical factors that influence the structure and dynamics of microbial ecosystems [21], while Miller et al. advocated for the use of metacommunity theory in explaining host-associated microbiome variation [22]. More recently, Kinnunen et al. implemented the community ecology framework to clarify potential determinants of invasion [23], and Verster and Borenstein proposed a competitive lottery model for the occupancy of microbial niches by different ecotypes [24].

The present work provides a conceptual framework for the phylogenetically constrained assembly of microbial communities. First, I discuss the observed microbial community and genomic characteristics on which the framework is grounded. Then, I enumerate a series of hypotheses and predictions that form the backbone of the framework and provide supporting evidence from the literature, as well as dedicated supporting results. I continue by considering the effect of ecosystem patchiness, social interactions, and sampling pitfalls on the model, and finally ponder on its limitations, implementation, and utility.

## Supporting microbial characteristics

### Traits and ecological function are, to some extent, phylogenetically conserved

Despite the pervasive horizontal gene transfer phenomenon among bacteria, tendency to gene loss, or convergent evolution, many traits are conserved across the microbial phylogeny (for a review, see [25]). Evolutionarily related microbial populations share more traits than expected from a random distribution along the phylogeny, and the depth of trait conservation correlates inversely with its complexity [26, 27].

The link between phylogenetic relatedness and ecological function or gene content similarity was further substantiated through the literature review of 990 microbial traits [28] or the correlation analysis of 16S rRNA phylogenetic marker genes and genomic content along the bacterial phylogeny [29]. Furthermore, a substantial agreement between evolutionary relatedness and nutritional requirements was found analyzing the metabolic networks of 478 species [30]. Significantly, the phylogenetic coherence of ecological traits was also observed over large phylogenetic distances [3]. Known concrete examples include the exclusive conservation within the *Vibrionaceae* of many ABC transporters and two-component systems likely related to niche space [31], methanogenesis in *Euryarchaeota* and acetoclastic methanogenesis in *Methanosarcinales* [32], oxygenic photosynthesis in *Cyanobacteria* [26], or the observed deep phylogenetic conservation of a particle-associated lifestyle in bathypelagic prokaryotes [33].

### Trait-based deterministic processes have a significant impact on community assembly

Community assembly is often probed in terms of two opposing theories [7]: neutral theory, where stochastic forces dominate assembly, and niche theory, where deterministic interactions between individuals, populations, and the environment determine community composition. Hubbell’s neutral theory [34] states that all species are ecologically equivalent, and stochastic processes (drift, dispersal, and speciation) govern community composition. Significantly, it provides null models for assessing the role of selection by comparing observed variation in community composition to that expected when community assembly is governed solely by stochastic processes [35]. On the other hand, niche theory states that deterministic factors (species traits, abiotic factors, and biotic interactions) determine community composition [36]. Here, advantageous trait combinations, allowing to effectively surpass abiotic filters and/or navigate biotic interactions, are selected in the ecosystem [37]. While the effect of biotic interactions in determining community structure remains comparatively underexplored (for a noteworthy exception, see [16]), there is a wealth of research supporting the importance of selection via abiotic factors (e.g., [38, 39]).

The niche-versus-neutral dichotomy framework has been surpassed thanks to a series of reports indicating that both neutral and deterministic processes play a simultaneous role in shaping community composition [40, 41]. Moreover, the use of Vellend’s community ecology framework supersedes the former, since it can account for both niche-based and neutral processes simultaneously shaping community structure [7]. Furthermore, recent studies have reported transitions between neutral and selective regimes in microbial communities [42]. For instance, the gut environment of both zebrafish and humans seems to be initially colonized through stochastic processes but compositional convergence ensues, a hallmark of deterministic processes [43, 44].

### Most microbial communities are phylogenetically clustered

Within most microbial communities, bacteria tend to co-occur with phylogenetically related populations more often than expected by chance [40, 45, 46], a phenomenon termed phylogenetic clustering or phylogenetic underdispersion. Less frequently, some communities display the opposite pattern, phylogenetic overdispersion, where co-occurring populations are less evolutionarily related than expected [47, 48]. Interestingly, the explanations provided for both patterns are grounded on the idea of a certain level of coherence between phylogeny and ecological function (see above); phylogenetic clustering is commonly linked to the presence of significant abiotic filtering in community assembly and supposedly arises from the existence of groups of related populations sharing a series of traits allowing them to surpass the abiotic filter [45, 49]. This idea is in line with the observation that genome composition and phylogeny drive co-occurrence patterns globally [27, 50]. On the other hand, phylogenetic overdispersion allegedly relates to biotic interactions; the existence of competitive exclusion between phylogenetically (ecologically) similar populations [45, 49] would lead to the co-occurrence of phylogenetically distant populations.

## A new microbial community assembly principle

Here, I synthesize the abovementioned facts that trait-based deterministic processes have a significant impact on microbial community assembly, that microbial communities most commonly show significant phylogenetic signal (clustering or overdispersion), and that microbial traits and ecological coherence are, to some extent, phylogenetically conserved, to produce a new community assembly principle.

Selection acts on particular combinations of traits, where such combinations may or may not show phylogenetic conservation. The only plausible explanation for the pervasive existence of phylogenetic signal in most microbial communities is the existence, within such communities, of prevalent combinations of abiotic and/or biotic factors selecting for particular combinations of traits showing phylogenetic conservation. Hence, I conceptualize selection as divisible into two niche categories: (i) niches whose occupancy requires a specific phylogenetically conserved set of traits (from now on “phylo-niches”) and (ii) niches whose occupancy requires specific sets of traits not showing strong phylogenetic conservation (from now on “non-phylo-niches”). It becomes necessary at this point to clarify that the notion “niche occupancy” is used in the present framework to signify the ability to persist in the ecosystem by surpassing a particular combination of abiotic and/or biotic filters.

From here, I derive the following predictions that form the basis of the framework. First, any microbial ecosystem can present phylo-niches and non-phylo-niches. Second, for each phylo-niche, there must be a discrete portion of the phylogeny (from now on a “phylogenetic core group,” or PCG) whose members share a phylogenetically conserved set of traits allowing the occupancy of their respective phylo-niche. Third, for each non-phylo-niche, there must be a group of microbial populations sharing a set of traits not showing phylogenetic conservation allowing the occupancy of their respective non-phylo-niche.

Thus, each instance of the same microbial ecosystem type should present populations from each PCG (occupying phylo-niches) and non-phylogenetic-core populations (occupying non-phylo-niches). Populations occupying each of both the phylo and non-phylo type niches should present a high degree of intra-group ecological coherence, due to their shared traits, and hence, their intra-group structure should be governed by neutral processes and likely show intra-group competition.

## Support from the literature and dedicated results

The assembly principle presented is well in line with results from recent works studying community assembly through different approaches. Lu et al. [51] evaluated the strength of community-environment relationships at different taxonomic resolutions in eight selected case studies and found that the variation in community structure explained by environmental parameters either increased or remained constant with broadening taxonomic resolution. This result led them to hypothesize the existence of overlapping ecological coherence at broader taxonomic resolutions, hence substantiating the idea that the niche-phylogeny relationship is not restricted to closely related populations.

Burns and co-workers [44] showed that throughout zebrafish development, certain gut bacterial OTU abundances significantly deviated from predictions under a neutral model and that the overall non-neutral partition of the dataset was phylogenetically clustered, leading them to hypothesize that potentially important taxa could be identified by their divergence from neutral distributions. More recently, Harris et al. [52] detected similar patterns in the human gut. Significantly, they carried out the analysis at different levels of taxonomic resolution and found that significant departure from predictions under a neutral model appeared at different taxonomic levels throughout the bacterial kingdom, which they took as plausible indication of breaking points of ecological overlap. These results and conclusion are clearly analogous to the abovementioned prediction of PCGs showing intra-group neutral dynamics and ecological coherence. Another evidence related to the existence of intra-phylo-niche neutrality is the observation that stochasticity drives the colonization success of an invading population when displacing closely related resident bacterial populations [53].

Russel et al. [54] analyzed 2211 pairs of species from 8 different environments and reported a clear inverse association between antagonism (growth inhibition) and phylogenetic distance, which agrees with intra-PCG competition. Also in line with this idea, Peay and co-workers [55] observed a strong positive correlation between priority effects (strong competition) [8] and phylogenetic relatedness in the assembly of nectar microbial communities. In line with the postulated existence of varying-depth PCGs with intra-group competition, Verster and Borenstein recently showed that various clades along the bacterial taxonomy show intra-group priority effects in the human gut microbiome [24].

Turning to a simpler host-associated microbial community, four preeminent *Lactobacillus* species appear to compete for the same niche in the vaginal microbiome of reproductive-age women [56]. These lactobacilli seemingly represent an appropriate example of a PCG; the group is present in all instances of the community (with exceptions, see “Discussion” section), its members are closely related, likely possess a phylogenetically conserved suite of traits allowing them to persist in the ecosystem and compete for the same niche. Most evident among such shared traits is the ability to consume α-amylase-cleaved [57] host-derived glycogen (selection related to what can be considered here as an abiotic filter) and produce lactate, lowering the pH which prevents the establishment of competitors (selection related to biotic interactions).

Recently, Goldford et al. [16] studied community assembly on a single carbon and energy source (either glucose, citrate, or leucine). Using ex situ cultivation of complex microbial communities derived from different natural habitats, they found that, for each compound, communities assembled into highly variable compositions at the shallowest phylogenetic level analyzed (16S rRNA gene exact sequence variants; ESVs). Nevertheless, the same family-level compositions arose for each compound despite the very diverse starting natural communities. The authors employed the term “family-level attractors” to describe the phenomenon and *hypothesized that taxonomic convergence might reflect selection by functions that are conserved at the family level*. Such selection was obviously enforced not only by abiotic factors (the synthetic media and culturing conditions employed) but also by biotic interactions, since they also showed that individual ESVs from the different taxa analyzed were able to independently grow on the substrate. The parallelism between their findings and hypothesis, and the predictions described above are evident; the “attractors” indicate the existence of phylo-niches, which in turn prompt the appearance of populations from each PCG (the dominant families) in all instances of each microbial ecosystem type. The PCGs in their more exhaustive glucose experiments related to the *Enterobacteriaceae* and *Pseudomonadaceae* families. The authors postulated that the emergence of such families could be related to competitive advantages associated with the uptake capabilities of their phosphotransferase system and adenosine triphosphate-binding cassette transporters, respectively. This conclusion links to the above prediction that PCGs share a phylogenetically conserved set of traits allowing them to surpass a particular combination of filters.

The assessment of compositional “cores” in microbial communities in terms of bacterial groups of varying phylogenetic depths has previously almost exclusively been attempted in terms of taxonomic assignments (e.g., [58]). However, taxonomic assignments are heavily biased towards well-sampled groups [59] and represent a coarse-grained stratification of the phylogenetic continuum. Moreover, within-group phylogenetic and gene content similarity of taxa in the same rank are not homogenous, and these values show extensive overlap between ranks [29]. Hence, the description of PCGs in terms of taxonomic assignments is not adequate when considering subsequent analyses and applications. I recently studied the human gut microbiome in terms of 16S rRNA gene OTUs present in all individuals, where such OTUs had been produced dynamically over a range of similarity clustering thresholds [60]. Through the analysis of comprehensive independent datasets [61, 62], I observed that the human gut microbiome indeed contained a preeminent compositional phylogenetic core, defined in terms of discrete units of varying depth along the bacterial phylogeny, whose members were present in all individuals studied, an adequate proxy for PCGs. Following the same strategy, I have later observed PCGs in all three microbial compartments of the rice root ecosystem, as well as within yet another large-cohort human gut microbiome dataset (Additional file 1).

## Second-tier: accounting for patchiness, biotic interactions, and higher-scale sampling

Before we can propose a community assembly model that can aspire to recapitulate the observed patterns of diversity in complex microbial ecosystems, we need to address a few additional factors; patchiness in microbial ecosystems, microbial social interactions, and scale-related sampling pitfalls. As recently reviewed by Cordero and Datta [63], microbial ecosystems most often consist of patches of strongly interacting dense microbial consortia, even in apparently well-mixed oligotrophic planktonic habitats [64]. Within these microscale patches, short cell to cell distances allow for efficient diffusion-mediated metabolite exchange, leading to strong biotic interactions with a significant influence on community structure and dynamics. For instance, competing or mutually antagonistic populations may exclude each other on a particular patch depending on the scale of the patch relative to the reach of the antagonistic effect, but can co-exist in the ecosystem when considering all patches. For example, in an activated granular sludge reactor, different genotypes of *Candidatus accumulibacter* excluded each other within each patch (granule), but coexisted in the metacommunity (reactor) [65]. Hence, the scale of sampling relative to that of the local community (see below) may influence the emergence of different phylogenetic signal patterns (over or underdispersion).

Another important aspect is the possible existence of strongly interacting species (SIS) [66] within the regional species pool. One possible origin for such SIS stems from the Black Queen Hypothesis [67], which relates to the evolution of dependency between populations through adaptive gene loss. Partially metabolically redundant populations may give rise, following different evolutionary trajectories of gene loss, to different co-evolved inter-dependent groups, whose members are not interchangeable but form functionally equivalent ensembles. On the other hand, colonizing/invading populations with different functional repertoires may exert local niche-modifying effects, strongly influencing community assembly, and producing functionally inequivalent local communities for identical abiotic environments ensembles. Thus, and as initially advanced by Gleason almost a century ago [68], the order of community assembly may impact its final structure due to biotic interactions, even under identical environmental conditions and regional species pool. In the context of within-patch (i.e., local) community assembly, the existence of such particular social interactions would drive alternative compositional states, which may be functionally equivalent or not. In this regard, Gibson and co-workers showed, using computer simulations, that different community types in the human gut microbiome (i.e., enterotypes) emerged only in the presence of different SIS combinations [66]. On the other hand, the effect of the niche modifying capabilities of particular populations on the development of different functionally inequivalent community structures has been recently demonstrated for the rumen environment [69].

Returning to the abovementioned Goldford et al.’s study featuring the emergence of *Enterobacteriaceae* and *Pseudomonadaceae* as PCGs when diverse natural communities were cultivated ex situ on glucose [16], the different experimental replicates contained alternative *Pseudomonadaceae*-affiliated ESVs. This behavior represents the simplest scenario of intra-PCG ecology; intra-group competition and dominance of a single PCG population. However, within the *Enterobacteriaceae*, the replicates presented either a *Klebsiella*-affiliated ESV or a guild consisting of variable compositions of ESVs affiliated to the *Enterobacter*, *Raoultella*, and/or *Citrobacter* genera. In this case, we find again an agreement with the prediction of intra-PCG competition. However, in the case of the *Enterobacteriaceae*, the intra-group structure was partitioned among two alternative structural states: either an ESV affiliated to *Klebsiella* or a variable guild of ESVs affiliated to three different genera. While it is not possible without further analysis to anticipate which of the abovementioned mechanisms is responsible for the phenomenon, it serves to demonstrate how the stochastic moiety of community assembly can result in biotic interactions with an impact on local community structure. Moreover, while the assembly principle postulated states that only particular populations can occupy each specific niche, in reality, either a single population or a functionally cohesive guild of populations can occupy each particular niche, provided that the guild as a whole presents the set of traits (phylogenetically conserved or not) required by the niche.

Finally, we need to take into account that collected environmental samples comprise multiple different abiotic microenvironments and local communities [63], with the noteworthy exception of a few known natural microbial communities such as granules in activated sludge bioreactors [65] or pink berries [70]. Hence, statistical associations and community composition inventories inferred from such coarse-grained samples only partially reflect local community assembly, being most likely biased by the relative preeminence of the different microenvironments in each particular sample, a phenomenon I term “higher-scale sampling.”

## A phylogenetically constrained assembly model

**Fig. 1:**
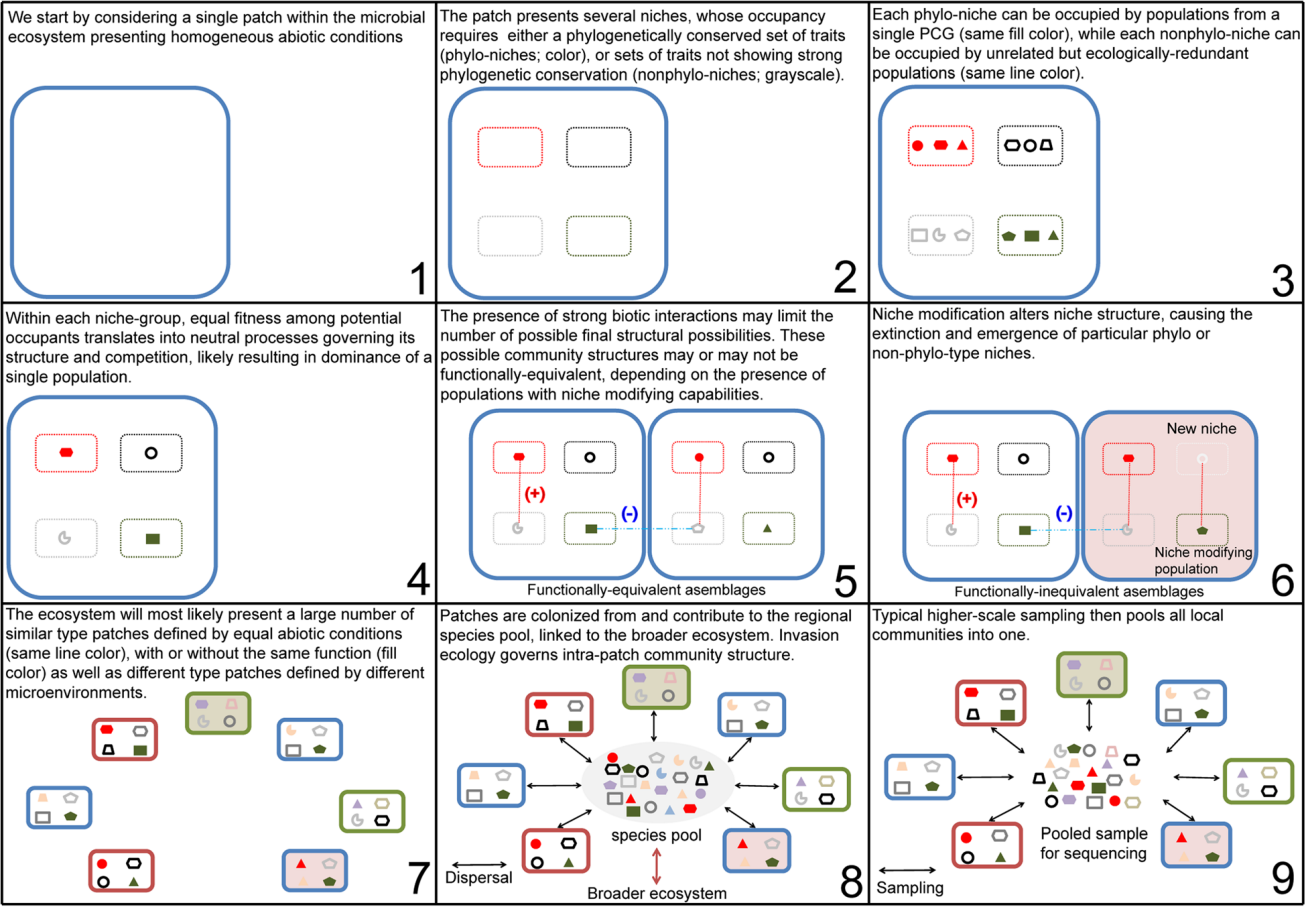
A phylogenetically constrained assembly model

Having conceptually split selection as acting on particular trait combinations showing either phylogenetic conservation (phylo-niches) or not (non-phylo-niches), and accounting for a second tier of effects related to patchiness, biotic interactions, and higher-scale sampling, I propose the following step-wise assembly model which aspires to recapitulate the observed patterns in microbial community composition. We start by considering a single patch (local community) within the microbial ecosystem presenting particular abiotic conditions (Fig. 1). The patch presents several potential niches, whose occupancy requires either a phylogenetically conserved set of traits (phylo-niches) or sets of traits not showing strong phylogenetic conservation (non-phylo-niches). Each phylo-niche can be occupied by populations from a single PCG, while each non-phylo-niche can be occupied by unrelated but ecologically redundant populations. Within each niche-group, equal fitness among potential occupants translates into neutral processes governing its structure, and intra-group competition, likely resulting in the dominance of a single population or particular spatial organizations. However, the sum of the populations occupying each niche should be non-neutral and governed by relative niche-size. The presence of strong biotic interactions may limit the number of possible final community types. These community types may or may not be functionally equivalent, depending on the presence of populations with niche modifying capabilities. Niche modification works by altering the patch’s niche structure, causing the extinction and emergence of particular niches, again subject to the abovementioned rules. Patches are colonized from and contribute to the regional species pool, which is in turn linked to the broader ecosystem. Stochastic events may result in different intra-niche and hence intra-patch community structures over time, or the extinction of a particular patch and the birth of a new patch. Moving away from the single patch, the ecosystem will most likely present a large number of similar type patches, defined by equal abiotic conditions, as well as different type patches defined by different abiotic conditions. Typically, higher-scale sampling then pools all local communities into one microbial community sample.

## Discussion

### The proposed assembly model recapitulates the characteristics most commonly observed in microbial communities

Microbial communities show remarkable functional stability despite large species turnover, a phenomenon here explained by intra-niche stochasticity among populations featuring functional coherence.

Microbial communities are highly diverse, show large species richness, and feature the coexistence of theoretically competing populations. These patterns arise from the pervasive existence of different patch assemblages and patch types in environmental samples as discussed by Cordero and Datta [63] (as well as by intra-niche stochasticity), which higher-scale sampling presents as pertaining to the same community.

Significantly, the presented model, built on the theoretical existence of phylo-niches, also explains the prevalent phylogenetic signal commonly observed in microbial communities, the presence of PCGs detected at different phylogenetic depths observed in different environments, and the link between invasion success and the presence in the resident community of closely related populations.

### The proposed model extends the community ecology framework to account for patchiness, higher-scale sampling, and phylogeny-related selection

With regard to the relation between the assembly model presented and Vellend’s community ecology synthesis, here, selection has been conceptually split into the existence of two niche categories depending on whether it acts on particular trait combinations showing phylogenetic conservation or not. In turn, drift and dispersal dictate niche occupancy by suitable populations. Moreover, historical contingencies related to the existence of SIS (with or without niche modifying capabilities), drift, and dispersal may affect the ecosystem’s patch structure, as explained above. Speciation is not explicitly accounted for in the model, which pertains mainly to community assembly, yet new genetic types are still subject to the proposed assembly rules. Finally, higher-scale sampling and abiotic microheterogeneity dictate that most analyzed microbial samples should be understood as representing a regional community formed by multiple local communities and abiotic patch types.

### The use of the proposed conceptual framework in the study of microbial communities provides various routes of implementation

**Fig. 2:**
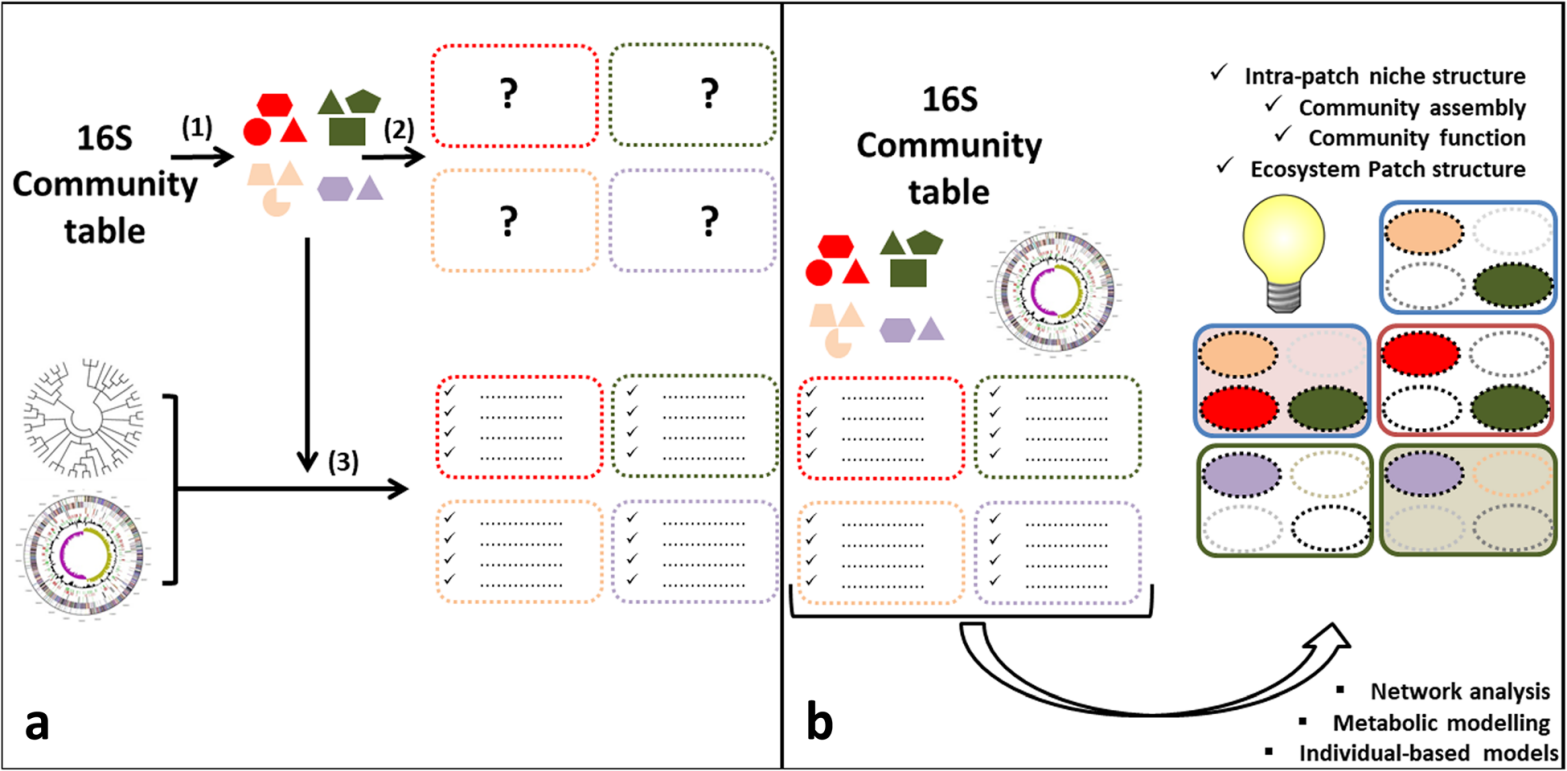
Framework implementation towards an increased understanding of microbial ecosystems. **a** Phylogenetic sequence analysis identifies PCGs from the 16S community table (1). The framework predicts the existence of a number of phylo-niches in the ecosystem (2). Bioinformatic analysis using PCG phylogeny and genomic databases illuminates phylo-niche characteristics (3). **b** The use of community composition, PCG structure, genomic databases, and pylo-niche characteristics to prime complementing network analysis, metabolic, and individual-based models will translate into an unprecedented understanding of the ecosystem, illuminating patch niche structure, assembly rules, and function, as well as ecosystem patch structure

The immediate practical value of the proposed framework rests on the fact that high-throughput inexpensive phylogenetic profiling of microbial community samples can be accomplished using 16S marker gene amplicon sequencing. From that point, PCGs detection and thus phylo-niche description in terms of sets of traits being selected can be accomplished using available bioinformatic resources. In this regard, microbial ecosystems of interest could be analyzed using the following general pipeline (Fig. 2): First, an appropriate number of samples from the ecosystem under study are analyzed by 16S amplicon sequencing, producing community abundance inventories. Then, phylogenetic sequence analysis identifies existing PCGs as the minimal portions of the phylogeny present in all samples. The framework predicts the existence of phylo-niches in the ecosystem for each detected PCG, related to the idea that members of each PCG share a phylogenetically conserved combination of genes allowing their occupancy of such niches. Thus, for each PCG, the joint bioinformatic analysis of its phylogeny and estimated core pan-genome provides information related to its members’ shared functionality, illuminating their ecological function. Importantly, the number of samples being analyzed should be high enough so as to properly capture stochasticity in intra-niche occupancy by suitable populations, allowing the proper delineation of PCGs.

Similar overall approaches have been carried out previously. For instance, members of the *Acidobacteria* are ubiquitous in soil environments. Recently, Eichorst et al. [71] compared available genomes from that phylum to identify features that could explain their high prevalence and ecophysiology in soils. Their results pinpointed a high frequency of high- and low-affinity oxygenases among the genomes, which they took as suggestive of the ability to grow at different oxygen gradients (a typical feature of the soil environment) being their strategy for success in terrestrial ecosystems. In this example, the implementation of the proposed framework would have detected other PCGs/phylo-niches in the ecosystem, as well as provided a phylogenetically sharper delineation of the *Acidobacteria*-affiliated group of populations prevalent in the ecosystem, which in turn would have provided a more relevant core pan-genome for analysis and description of their respective phylo-niche.

Additionally, the results from both the PCG detection/phylo-niche description approach and the assembly rules proposed can be used to prime network analysis, metabolic modeling, and individual-based models. While I cannot advance a general pipeline as above, it seems reasonable to argue that a greatly increased knowledge of the ecology (intra-group structure) and ecophysiology (niche description) for a substantial fraction of the community (i.e., populations belonging to PCGs) should improve our ability to understand the role of non-PCG populations in the ecosystem, for instance, by reducing the complexity of the system collapsing intra-PCG populations as ecologically equivalent entities. On the other hand, the assembly rules proposed could be used in community assembly simulation and individual-based models. Finally, it should be more commonly acknowledge that co-occurrence networks will, in most cases, bear mixed signals; co-occurrence may arise from positive biotic interactions or from the shared ability to thrive in a particular abiotic patch type.

### The framework presents various limitations

The usefulness of the proposed approach will be affected by the ability of the available sequencing depth to adequately sample the different patch types in the ecosystem. It will also vary from ecosystem to ecosystem, mainly governed by the proportion of phylo to non-phylo-niches. For instance, the framework may not be too effective in the study of the microbial community associated with the green macroalga *Ulva australis*, as it was reported that *U. australis* samples presented very high phylogenetic variability [72]. The opposite may be exemplified by the human gut microbiota, where PCGs pooled abundances accounted for a large fraction of the total community [60]. Furthermore, the framework’s utility will be affected by the heterogeneity of the abiotic conditions relative to the actual sample scale.

The framework is grounded on the theoretical existence of similar phylo-niches in all(most) instances of the ecosystem under study. However, while such instances may present a similar abiotic environment, the activity of niche modifying populations may impact niche structure, as mentioned earlier. When the impact is sufficiently important, it translates into different microbial regimes for the same initial environment. Also, different microbial regimes for the same ecosystem may be due to hidden abiotic differences. For example, different microbial regimes have been reported in the rumen for the same abiotic environment [69], in the human gut of healthy and IBD suffering individuals (different abiotic environments), or in the human vagina, where, out of five main community types detected one was not dominated by lactobacilli (unknown causes) [56]. Hence, the proposed framework should be applied to each different microbial regime independently. In this regard, an initial exploration of community compositions should easily reveal the existence of different microbial regimes and partition the dataset accordingly. The results of applying the framework to each microbial regime independently can be later compared, providing an increased understanding of the microbial ecosystem. For similar reasons, care should be taken when analyzing microbial ecosystems presenting strong community succession patterns where selection factors greatly vary from stage to stage.

Notably, the 16S rRNA gene presents low resolution at the highest and lowest phylogenetic depths [73], and thus the present framework cannot be used with that gene to discern selection patterns related to such depths. Finally, an anticipated critique of the framework relates to the in many respects more effective use of metagenomics/metatranscriptomics to study microbial communities. It seems clear that shotgun metagenomic data allows for a much higher resolution in the analysis of community assembly and function than the proposed framework, but only if sufficient sample replication and sequencing depth is available. To date, this is arguably only the case of the human gut ecosystem. Hence, given the need to analyze many samples to properly study community assembly in an ecosystem, the myriad of economically and/or scientifically important microbial ecosystems on Earth, and taking into account costs (financial, human resources-related, and computational), the proposed framework offers an inexpensive avenue to gain insights into Earth’s microbial ecosystems.

## Conclusion

The common patterns of microbial community organization observed in nature suggest the existence of fundamental community assembly rules. In this regard, the lack of an adequate microbial community assembly model is hindering our ability to understand and manage microbial ecosystems, hampering our capacity to improve a plethora of environmental, agricultural, and health-related practices. Here, I have presented a community assembly conceptual framework built on well-known microbial community and genomic characteristics, supported by the recent literature and dedicated results, and able to recapitulate the characteristics most commonly observed in microbial communities. By pinpointing the theoretical existence of phylo-niches, the framework has the potential to increase our understanding of microbial ecosystems; PCGs’ identification is inexpensive and relatively straightforward using 16S sequencing, and subsequent bioinformatic analysis of their phylogeny and estimated core pan-genomes should provide valuable information on their ecophysiology and niche characteristics. Providing such a priori information for a significant portion of the community should greatly enhance the capacity of complementing analyses to further explain microbial community assembly and function. Thus, the use of the proposed framework should represent a leap forward in our ability to understand, manage, or remediate microbial ecosystems.

The prediction that microbial communities should present PCGs has so far been substantiated by the analysis of various datasets and ecosystems. Future work should study to which degree the different populations within each PCG present shared ecological functionality, and how such particular functionality relates to the overall ecosystem’s function, and the ability of the group to occupy a particular niche within the ecosystem. It should also be assessed if these intra-core group populations exhibit competitive interactions, as could be expected by their theorized ecological redundancy. Lastly, alternative community assembly models should adequately explain the observed existence of extensive phylogenetic signal in microbial communities, the presence of PCGs in analyzed microbial ecosystems, and the link between invasion success and the presence in the resident community of closely related populations.

## Supplementary information

**Additional file 1.** Supplementary Materials to “A conceptual framework for the phylogenetically-constrained assembly of microbial communities.”

## Data Availability

The datasets analyzed during the current study are available from their original source (Additional file 1). Additional result files and scripts are available from the corresponding author on reasonable request.

## References

[1] Falkowski PG, Fenchel T, Delong EF (2008). The microbial engines that drive Earth’s biogeochemical cycles. Science.

[2] Vellend M (2010). Conceptual synthesis in community ecology. Q Rev Biol.

[3] Philippot L, Andersson SG, Battin TJ, Prosser JI, Schimel JP, Whitman WB (2010). The ecological coherence of high bacterial taxonomic ranks. Nat Rev Microbiol.

[4] Brodie JF, Redford KH, Doak DF (2018). Ecological function analysis: incorporating species roles into conservation. Trends Ecol Evol.

[5] Wilson DS (1992). Complex interactions in metacommunities, with implications for biodiversity and higher levels of selection. Ecology.

[6] Leibold MA, Holyoak M, Mouquet N, Amarasekare P, Chase JM, Hoopes MF (2004). The metacommunity concept: a framework for multi-scale community ecology. Ecol Lett.

[7] Nemergut DR, Schmidt SK, Fukami T, O'Neill SP, Bilinski TM, Stanish LF (2013). Patterns and processes of microbial community assembly. Microbiol Mol Biol Rev.

[8] Alford RA, Wilbur HM (1985). Priority effects in experimental pond communities: competition between Bufo and Rana. Ecology.

[9] Violle C, Navas M-L, Vile D, Kazakou E, Fortunel C, Hummel I (2007). Let the concept of trait be functional!. Oikos.

[10] Zhou J, Ning D (2017). Stochastic community assembly: does it matter in microbial ecology?. Microbiol Mol Biol Rev.

[11] Graham EB, Knelman JE, Schindlbacher A, Siciliano S, Breulmann M, Yannarell A (2016). Microbes as engines of ecosystem function: when does community structure enhance predictions of ecosystem processes?. Front Microbiol.

[12] Thompson LR, Sanders JG, McDonald D, Amir A, Ladau J, Locey KJ (2017). A communal catalogue reveals Earth’s multiscale microbial diversity. Nature.

[13] Dessaux Y, Grandclément C, Faure D (2016). Engineering the rhizosphere. Trends Plant Sci.

[14] Zmora N, Zeevi D, Korem T, Segal E, Elinav E (2016). Taking it personally: personalized utilization of the human microbiome in health and disease. Cell Host Microbe.

[15] Konopka A, Lindemann S, Fredrickson J (2015). Dynamics in microbial communities: unraveling mechanisms to identify principles. ISME J.

[16] Goldford JE, Lu N, Bajić D, Estrela S, Tikhonov M, Sanchez-Gorostiaga A (2018). Emergent simplicity in microbial community assembly. Science.

[17] Hanson CA, Fuhrman JA, Horner-Devine MC, Martiny JB (2012). Beyond biogeographic patterns: processes shaping the microbial landscape. Nat Rev Microbiol.

[18] Gilbert JA, Lynch SV (2019). Community ecology as a framework for human microbiome research. Nat Med.

[19] Stegen JC, Lin X, Fredrickson JK, Chen X, Kennedy DW, Murray CJ (2013). Quantifying community assembly processes and identifying features that impose them. ISME J.

[20] Evans S, Martiny JB, Allison SD (2017). Effects of dispersal and selection on stochastic assembly in microbial communities. ISME J.

[21] Stegen JC, Bottos EM, Jansson JK (2018). A unified conceptual framework for prediction and control of microbiomes. Curr Opin Microbiol.

[22] Miller ET, Svanback R, Bohannan BJM (2018). Microbiomes as metacommunities: understanding host-associated microbes through metacommunity ecology. Trends Ecol Evol.

[23] Kinnunen M, Dechesne A, Proctor C, Hammes F, Johnson D, Quintela-Baluja M (2016). A conceptual framework for invasion in microbial communities. ISME J.

[24] Verster AJ, Borenstein E (2018). Competitive lottery-based assembly of selected clades in the human gut microbiome. Microbiome.

[25] Martiny JB, Jones SE, Lennon JT, Martiny AC (2015). Microbiomes in light of traits: a phylogenetic perspective. Science.

[26] Martiny AC, Treseder K, Pusch G (2013). Phylogenetic conservatism of functional traits in microorganisms. ISME J.

[27] Tamames J, Sánchez PD, Nikel PI, Pedrós-Alió C (2016). Quantifying the relative importance of phylogeny and environmental preferences as drivers of gene content in prokaryotic microorganisms. Front Microbiol.

[28] Goberna M, Verdu M (2016). Predicting microbial traits with phylogenies. ISME J.

[29] Parras-Moltó M, Aguirre de Cárcer, D. Assessment of phylo-functional coherence along the bacterial phylogeny and taxonomy. bioRxiv. 2019. p. 795914. 10.1038/s41598-021-87909-1 PMC8050241 33859339

[30] Borenstein E, Kupiec M, Feldman MW, Ruppin E (2008). Large-scale reconstruction and phylogenetic analysis of metabolic environments. Proc Natl Acad Sci U S A.

[31] Vitulo N, Vezzi A, Romualdi C, Campanaro S, Valle G (2007). A global gene evolution analysis on Vibrionaceae family using phylogenetic profile. BMC Bioinformatics.

[32] Lang K, Schuldes J, Klingl A, Poehlein A, Daniel R, Brunea A (2015). New mode of energy metabolism in the seventh order of methanogens as revealed by comparative genome analysis of “Candidatus methanoplasma termitum”. Appl Environ Microbiol.

[33] Salazar G, Cornejo-Castillo FM, Borrull E, Diez-Vives C, Lara E, Vaque D (2015). Particle-association lifestyle is a phylogenetically conserved trait in bathypelagic prokaryotes. Mol Ecol.

[34] Hubbell SP (2006). Neutral theory and the evolution of ecological equivalence. Ecology.

[35] Rosindell J, Hubbell SP, Etienne RS (2011). The unified neutral theory of biodiversity and biogeography at age ten. Trends Ecol Evol.

[36] Fargione J, Brown CS, Tilman D (2003). Community assembly and invasion: an experimental test of neutral versus niche processes. Proc Natl Acad Sci.

[37] Schmidt VT, Smith KF, Melvin DW, Amaral-Zettler LA (2015). Community assembly of a euryhaline fish microbiome during salinity acclimation. Mol Ecol.

[38] Powell JR, Karunaratne S, Campbell CD, Yao H, Robinson L, Singh BK (2015). Deterministic processes vary during community assembly for ecologically dissimilar taxa. Nat Commun.

[39] Lozupone CA, Knight R (2007). Global patterns in bacterial diversity. Proc Natl Acad Sci U S A.

[40] Stegen JC, Lin X, Konopka AE, Fredrickson JK (2012). Stochastic and deterministic assembly processes in subsurface microbial communities. ISME J.

[41] Zhou J, Deng Y, Zhang P, Xue K, Liang Y, Van Nostrand JD (2014). Stochasticity, succession, and environmental perturbations in a fluidic ecosystem. Proc Natl Acad Sci.

[42] Cira NJ, Pearce MT, Quake SR (2018). Neutral and selective dynamics in a synthetic microbial community. Proc Natl Acad Sci U S A.

[43] Guittar J, Shade A, Litchman E (2019). Trait-based community assembly and succession of the infant gut microbiome. Nat Commun.

[44] Burns AR, Stephens WZ, Stagaman K, Wong S, Rawls JF, Guillemin K (2016). Contribution of neutral processes to the assembly of gut microbial communities in the zebrafish over host development. ISME J.

[45] Horner-Devine MC, Bohannan BJ (2006). Phylogenetic clustering and overdispersion in bacterial communities. Ecology.

[46] Bryant JA, Lamanna C, Morlon H, Kerkhoff AJ, Enquist BJ, Green JL (2008). Colloquium paper: microbes on mountainsides: contrasting elevational patterns of bacterial and plant diversity. Proc Natl Acad Sci U S A.

[47] Thompson JR, Pacocha S, Pharino C, Klepac-Ceraj V, Hunt DE, Benoit J (2005). Genotypic diversity within a natural coastal bacterioplankton population. Science.

[48] Chaffron S, Rehrauer H, Pernthaler J, von Mering C (2010). A global network of coexisting microbes from environmental and whole-genome sequence data. Genome Res.

[49] Webb CO, Ackerly DD, McPeek MA, Donoghue MJ (2002). Phylogenies and community ecology. Annu Rev Ecol Syst.

[50] Kamneva OK (2017). Genome composition and phylogeny of microbes predict their co-occurrence in the environment. PLoS Comput Biol.

[51] Lu HP, Yeh YC, Sastri AR, Shiah FK, Gong GC, Hsieh CH (2016). Evaluating community-environment relationships along fine to broad taxonomic resolutions reveals evolutionary forces underlying community assembly. ISME J.

[52] Harris K, Parsons TL, Ijaz UZ, Lahti L, Holmes I, Quince C (2017). Linking statistical and ecological theory: Hubbell’s unified neutral theory of biodiversity as a hierarchical Dirichlet process. Proc IEEE.

[53] Kinnunen M, Dechesne A, Albrechtsen HJ, Smets BF (2018). Stochastic processes govern invasion success in microbial communities when the invader is phylogenetically close to resident bacteria. ISME J.

[54] Russel J, Roder HL, Madsen JS, Burmolle M, Sorensen SJ (2017). Antagonism correlates with metabolic similarity in diverse bacteria. Proc Natl Acad Sci U S A.

[55] Peay KG, Belisle M, Fukami T (2012). Phylogenetic relatedness predicts priority effects in nectar yeast communities. Proc Biol Sci.

[56] Ravel J, Gajer P, Abdo Z, Schneider GM, Koenig SSK, McCulle SL (2011). Vaginal microbiome of reproductive-age women. Proc Natl Acad Sci.

[57] Spear GT, French AL, Gilbert D, Zariffard MR, Mirmonsef P, Sullivan TH (2014). Human alpha-amylase present in lower-genital-tract mucosal fluid processes glycogen to support vaginal colonization by Lactobacillus. J Infect Dis.

[58] Falony G, Joossens M, Vieira-Silva S, Wang J, Darzi Y, Faust K (2016). Population-level analysis of gut microbiome variation. Science.

[59] Beiko RG (2015). Microbial malaise: how can we classify the microbiome?. Trends Microbiol.

[60] Aguirre de Cárcer D (2018). The human gut pan-microbiome presents a compositional core formed by discrete phylogenetic units. Sci Rep.

[61] Goodrich JK, Waters JL, Poole AC, Sutter JL, Koren O, Blekhman R (2014). Human genetics shape the gut microbiome. Cell.

[62] Yatsunenko T, Rey FE, Manary MJ, Trehan I, Dominguez-Bello MG, Contreras M (2012). Human gut microbiome viewed across age and geography. Nature.

[63] Cordero OX, Datta MS (2016). Microbial interactions and community assembly at microscales. Curr Opin Microbiol.

[64] Azam F, Malfatti F (2007). Microbial structuring of marine ecosystems. Nat Rev Microbiol.

[65] Leventhal GE, Boix C, Kuechler U, Enke TN, Sliwerska E, Holliger C (2018). Strain-level diversity drives alternative community types in millimetre-scale granular biofilms. Nat Microbiol.

[66] Gibson TE, Bashan A, Cao HT, Weiss ST, Liu YY (2016). On the origins and control of community types in the human microbiome. PLoS Comput Biol.

[67] Morris JJ, Lenski RE, Zinser ER (2012). The Black Queen Hypothesis: evolution of dependencies through adaptive gene loss. MBio.

[68] Gleason HA (1927). Further views on the succession-concept. Ecology.

[69] Shaani Y, Zehavi T, Eyal S, Miron J, Mizrahi I (2018). Microbiome niche modification drives diurnal rumen community assembly, overpowering individual variability and diet effects. ISME J.

[70] Wilbanks EG, Jaekel U, Salman V, Humphrey PT, Eisen JA, Facciotti MT (2014). Microscale sulfur cycling in the phototrophic pink berry consortia of the Sippewissett Salt Marsh. Environ Microbiol.

[71] Eichorst SA, Trojan D, Roux S, Herbold C, Rattei T, Woebken D (2018). Genomic insights into the Acidobacteria reveal strategies for their success in terrestrial environments. Environ Microbiol.

[72] Burke C, Steinberg P, Rusch D, Kjelleberg S, Thomas T (2011). Bacterial community assembly based on functional genes rather than species. Proc Natl Acad Sci U S A.

[73] Janda JM, Abbott SL (2007). 16S rRNA gene sequencing for bacterial identification in the diagnostic laboratory: pluses, perils, and pitfalls. J Clin Microbiol.

