# Supplementary material for: A conceptual framework for the phylogenetically constrained assembly of microbial communities
Source: Microbiome. 2019 Oct 30;7:142. doi: 10.1186/s40168-019-0754-y (PMC6822436; doi:10.1186/s40168-019-0754-y)
Supplement: Supplementary file 1 — Additional file 1. Supplementary Materials to “A conceptual framework for the phylogenetically-constrained assembly of microbial communities.” [file 40168_2019_754_MOESM1_ESM.docx]

**Supplementary Materials to “A conceptual framework for the phylogenetically-constrained assembly of microbial communities”**

Daniel Aguirre de Cárcer^1*^

^1^Departamento de Biología, Universidad Autónoma de Madrid, Madrid, Spain.

**SUPPLEMENTARY METHODS**

**Datasets.** Two sets of 16S rRNA gene sequences, obtained using primer pair F515-R806 targeting the V4 hypervariable region, were employed; FlemishGut [^1^](#_ENREF_1) (Belgium residents, Stool samples. Illumina MiSeq, 2x250bp sequences), and Rice [^2^](#_ENREF_2) (rhizosphere, rizoplane and endosphere samples from rice roots. Illumina MiSeq, 2x250bp sequences). The datasets analyzed during the current study are available from their original source.

**Sequence processing.** The same procedures were carried out for both datasets. QIIME (v1.9.1) [^3^](#_ENREF_3) scripts were employed during sequence processing. First, each dataset was subsampled to its maximum common depth. Then, chimeric sequences were identified (*usearch61*) and later removed. This procedure yielded 8.383 and 19.000 sequences per sample for FlemishGut (873 individuals) and Rice (371 samples) datasets, respectively. Dynamic OTU clustering and core OTU detection were carried-out as described by Aguirre de Cárcer [^4^](#_ENREF_4).

**SUPPLEMENTARY RESULTS**

| **Supplementary Table 1. Summary of Phylogenetic Cores**. | |
| --- | --- |
| **Dataset** | **Phylogenetic core groups (PCGs) per clustering threshold** |
| *TwinsUK* | 94**^1^**,92**^1^**,91**^2^**,90**^2^**,89**^1^**,88**^1^**,87**^3^**,85**^2^**,80**^1^** |
| *Global* | 97**^17^**,95**^3^**,93**^1^**,92**^4^**,91**^10^**,90**^16^**,89**^7^**,88**^18^**,87**^10^**,86**^12^**,85**^14^**,84**^13^**,83**^9^**,82**^5^**,81**^11^**, 80**^6^**, 79**^1^**,78**^3^**,76**^1^**,75**^1^** |
| **FlemishGut** | **97^2^, 95^1^, 93^1^, 92^2^, 87^3^, 86^1^, 84^2^, 83^3^, 82^1^, 77^1^** |
| **Rhizosphere** | **97^47^, 96^2^, 95^1^, 94^1^, 93^3^, 92^6^, 91^18^, 90^12^, 89^7^, 88^14^, 87^13^, 86^12^, 85^20^, 84^18^, 83^18^, 82^13^, 81^18^, 80^6^, 79^8^, 78^12^, 77^9^, 76^8^, 75^6^** |
| **Rhizoplane** | **97^32^, 96^1^, 95^1^, 94^2^, 93^1^, 92^2^, 91^5^, 90^6^, 89^3^, 88^8^, 87^3^, 86^7^, 85^7^, 84^9^, 83^13^, 82^15^, 81^11^, 80^8^, 79^6^, 78^9^, 77^7^, 76^6^, 75^5^** |
| **Endosphere** | **97^14^, 94^1^, 90^1^, 89^1^, 88^2^, 87^1^, 86^2^, 84^3^, 83^1^, 82^6^, 81^3^, 80^2^, 79^2^, 78^2^, 77^3^, 76^5^, 75^3^** |
| PCGs per clustering threshold; Numbers represent similarity clustering thresholds (x10^-2^) were OTUs with 100% prevalence were detected (proxy for PCG), and superscript values indicate the number of such OTUs observed for each threshold. **Bold**; results from this study. *Italics*; results from [^4^](#_ENREF_4). | |

**METHODOLOGICAL DISCUSSION**

The phylogenetic core approach described by Aguirre de Cárcer [^4^](#_ENREF_4) employed 16S rRNA sequence clusters obtained at different similarity thresholds. The rationale behind the strategy is that each OTU serves as proxy to a phylogenetic group, where the similarity threshold provides a representation of the lineage´s depth. In this regard, while it is commonly accepted that OTUs may not convey exact phylogenetic coherence [^5^](#_ENREF_5), they are overwhelmingly employed as descriptors of microbial diversity. However, the use of OTUs has its weaknesses, mainly related to the lack of true transitivity in the clustering of 16S sequences. For instance, it is not known which clustering method better preserves the phylogenetic relationships among sequences [^6^](#_ENREF_6), and the use of different algorithms may translate into the detection of phylogenetic cores with slightly differing compositions. Also, differences in initial seeding between clustering runs could produce similar effects. To remediate these issues, OTUs could be substituted by nodes in a phylogenetic tree, which should provide an enhanced phylogenetic resolution and precision of the core groups. On the other hand, the definition of core OTUs as “present in all samples” represents an effective, yet overly restrictive, heuristic. Future applications of the proposed framework could include enhanced definitions based on more sophisticated mathematical approaches or possibly based on the use of neutral models [^7^](#_ENREF_7)^,^[^8^](#_ENREF_8).

**1.**Falony, G. *et al.* *Science* 352, 560-564, (2016).

**2.**Edwards, J. *et al.* *Proceedings of the National Academy of Sciences* 112, E911, (2015).

**3.**Caporaso, J. G. *et al.* *Nat Meth* 7, 335-336, (2010).

**4.**Aguirre de Cárcer, D. *Scientific Reports* 8, 14069, (2018).

**5.**Koeppel, A. F. *et al.* *Nucleic Acids Res* 41, 5175-5188, (2013).

**6.**Schloss, P. D. *mSystems* 1, e00027-00016, (2016).

**7.**Burns, A. R. *et al.* *ISME J* 10, 655-664, (2016).

**8.**Harris, K. *et al.* *Proceedings of the IEEE* 105, 516-529, (2017).
